# Real-time monitoring of wavelet-based neurovascular coupling in neonates with hypoxic ischemic encephalopathy using an hourly time window

**DOI:** 10.1117/1.NPh.12.3.035011

**Published:** 2025-09-09

**Authors:** Soheila Norasteh, Hanli Liu, Srinivas Kota, Yu-Lun Liu, Rong Zhang, Lina F. Chalak

**Affiliations:** a University of Texas at Arlington, Department of Bioengineering, Arlington, Texas, United States; b UT Southwestern Medical Center and Children’s Medical Center, Division of Neonatal-Perinatal Medicine, Department of Pediatrics, Dallas, Texas, United States; c UT Southwestern Medical Center, Peter O’Donnell Jr. School of Public Health, Dallas, Texas, United States; d UT Southwestern Medical Center, Department of Neurology, Dallas, Texas, United States

**Keywords:** hypoxic ischemic encephalopathy, neurovascular coupling, wavelet transform coherence, amplitude-integrated electroencephalogram, cerebral oxygenation, dynamic neurovascular coupling

## Abstract

**Significance:**

Real-time monitoring of neurovascular coupling (NVC) is crucial for early diagnosis and effective treatment strategies in neonates with hypoxic ischemic encephalopathy (HIE). In our previous studies, the NVC of neonates with HIE was determined using wavelet transform coherence (WTC) between the amplitude-integrated electroencephalogram (aEEG) and regional cerebral oxygen saturation (${\mathrm{SctO}}_{2}$) using a post-acquisition analysis.

**Aim:**

We propose a time-resolved WTC analysis, providing a real-time analysis tool that facilitates immediate and continuous evaluation of cerebral hemodynamics and neuronal activity.

**Approach:**

The real-time WTC framework employs a progressive zero-padding strategy with incremental temporal data integration. Initial analysis preserves 4 h of $\mathrm{aEEG}/{\mathrm{SctO}}_{2}$ data while zero-padding 16 h to maintain a 20-h window. This enables calculation of time-resolved significant coherence (trSC) at time 2 h (1- to 2-h window) within the 20- to 150-min scale range. The system subsequently advances hourly, preserving an additional hour of acquired data while proportionally reducing zero-padding. This cascading approach continues until full 20-h data preservation, with final trSC calculations targeting time 18 h (17- to 18-h window).

**Results:**

We included 55 neonates with mild to severe HIE, the time-scale maps of which were obtained using both post-acquisition and real-time WTC analysis methods. Accordingly, trSC curves within the 20- to 150-min wavelet scale were statistically compared between the two methods using a linear mixed-effects model. There was no significant difference in trSC results between the two methods (p=0.159). In addition, NVC was significantly lower in the moderate to severe HIE group compared with the mild HIE group at hours 3 and 4 (p<0.01).

**Conclusions:**

We demonstrated the feasibility of real-time dynamic WTC analysis for dynamic NVC in newborns with HIE, providing a potential bedside tool for the early detection of brain abnormalities.

## 1 Introduction

Hypoxic ischemic encephalopathy (HIE) is a critical condition resulting from reduced oxygen and blood flow to the brain, affecting neonates worldwide. It is a leading cause of neurodevelopmental impairment, long-term disability, and neonatal mortality.^1^^,^^2^ A major clinical challenge in managing HIE lies in identifying the severity within the narrow therapeutic window for interventions, such as therapeutic hypothermia (TH).^3^^–^^5^ Neonatal HIE manifests after birth with encephalopathy of varying severity and potentially lifelong impairment.^6^^,^^7^ Current diagnostic methods rely heavily on neurological examination within 6 h of life, which is not practical for repeated assessments to monitor evolving encephalopathy. Neonates with mild HIE were not included in trials initially as evolving encephalopathy was difficult to identify soon after birth.^8^ Recent data in neonates with mild, untreated HIE revealed abnormal cognitive Bayley scores at 2 years and beyond.^9^^–^^12^ Importantly, the severity of secondary energy failure (rather than the clinical classification) is directly associated with abnormal outcomes and is the target of therapies.^13^ Quantitative biomarkers are needed to provide mechanistic insight into the severity/timing of injury of mild HIE. Our group has successfully developed a novel wavelet analytical platform that integrates bedside electroencephalogram (EEG) and near-infrared spectroscopy (NIRS) data to quantify neurovascular coupling (NVC) during TH for moderate to severe HIE.^14^ The platform effectively predicted persistent encephalopathy severity, MRI injury, and 2-year Bayley outcomes.^14^^–^^16^ The ability to identify within the first 6 h of age a subgroup of HIE at high risk of injury who were not diagnosed to have moderate or severe HIE is critical for planning neuroprotection studies in this understudied population.^17^^,^^18^

The neurovascular unit, which consists of neurons, astrocytes, endothelial cells of the blood–brain barrier, myocytes, pericytes, and components of the extracellular matrix, is essential for maintaining cerebral circulation homeostasis and facilitating the delivery of oxygen and nutrients to the brain.^19^^–^^21^ Enhancing the ability to assess NVC at the bedside in early hours of life in real time is vital for improving early detection and intervention strategies for at-risk neonates. Our recent studies have highlighted the methodology, feasibility, and clinical significance of quantifying NVC in the early hours of newborns using wavelet transform coherence (WTC) to associate EEG signals with cerebral tissue oxygen saturation (${\mathrm{SctO}}_{2}$) measured using NIRS.^14^^,^^15^^,^^22^ The WTC is a robust time–frequency analysis technique designed to evaluate the coherence between two time series across temporal and frequency scales. NVC can be assessed by applying WTC to amplitude-integrated electroencephalogram (aEEG) and regional ${\mathrm{SctO}}_{2}$ signals.^22^^,^^23^

Up to date, WTC has been applied to aEEG and ${\mathrm{SctO}}_{2}$ signals recorded over a certain duration, with analysis performed only after the completion of data acquisition.^15^^,^^22^^,^^24^ Although this post-acquisition analysis provides valuable NVC information, it presents a significant limitation in clinical scenarios where timely decision-making is crucial; therefore, real-time monitoring is key to success. For instance, neuroprotective interventions, such as TH, must be initiated within the first 6 h of life to prevent or mitigate the progression of encephalopathy.^5^ To overcome the limitations of conventional post-acquisition analyses used previously, this paper presents a novel dynamic analysis method to enable real-time assessment of NVC in the early hours of life in newborns. Specifically, our methodology, referred to as dynamic WTC (dWTC), is based on an empirical approach using a zero-padding technique for a pre-selected or fixed acquisition period (e.g., 20 h) to expand the length and width of the time–frequency window. The strategy of having a fixed time–frequency window size across the entire data collection duration facilitates consistent and required wavelet scales that can be obtained only with a long period of data acquisition. After statistical testing and confirmation of the new dWTC analysis, we present a useful and reliable clinical tool for the real-time assessment of NVC in neonates with HIE.

## 2 Materials and Methods

### 2.1 Study Population and Measurement Protocol

Full-term newborns (≥35 weeks’ gestational age) diagnosed with HIE and admitted to the neonatal intensive care unit at Parkland Health, Dallas, Texas, United States, between 2018 and 2025 were recruited for this prospective cohort study. The study (STU 022015-104) was approved by the institutional review board at the University of Texas Southwestern Medical Center, and parental consent was obtained before enrollment. The inclusion criteria were as follows: (1) a history of an acute perinatal event (e.g., placental abruption, cord prolapse, or decreased fetal heart rate), (2) umbilical cord arterial pH or arterial blood gas pH at <1  h postnatal age of ≤7.0 or a base deficit ≥15  mmol/L, and (3) clinical signs of encephalopathy. Exclusion criteria included any genetic or congenital conditions, a head circumference <30  cm, or a birth weight <1800  g as these factors could influence study outcomes.

### 2.2 Encephalopathy Severity Classification

A modified Sarnat exam^9^ was conducted by trained and certified attending physicians to assess the severity of HIE using the NICHD criteria.^25^ The total Sarnat Score (TSS) was calculated by adding the scores from all six categories, which ranges from 0 (normal in all categories) to 18 (severe in all categories).^9^^,^^17^ The clinical grade of HIE was classified as mild, moderate, or severe based on the number of abnormalities in the Sarnat exam. If there were an equal number of abnormalities, the grade was determined by the level of consciousness. The TSS for enrolled study participants was obtained from electronic health records.

### 2.3 EEG and NIRS Data Preprocessing

For the cohort recruited between 2018 and 2019, noninvasive EEG electrodes (Fz, C3, Cz, C4, P3, P4, O1, and O2) were placed on the newborns’ scalps according to the modified 10 to 20 international system for neonates. For the cohort recruited between 2023 and 2025, EEG data were collected using 19 electrodes (C3, C4, Cz, F3, F4, F7, F8, Fp1, Fp2, Fz, O1, O2, P3, P4, P7, P8, Pz, T7, and T8) placed at standard positions in accordance with the modified 10 to 20 international system for neonates. EEG signals were referenced to the mid-parietal electrode (Pz), with Pz used as the ground during data acquisition. Data were acquired at a sampling rate of 256 Hz using the Nihon Kohden system (Irvine, California, United States). ${\mathrm{SctO}}_{2}$ was simultaneously measured for each neonate’s center of forehead using an INVOS 4100–5100 oximeter (Somanetics, Troy, Michigan, United States) equipped with neonatal sensors, recording at a sampling rate of 0.21 or 0.03 Hz.

To enable the simultaneous acquisition of EEG and ${\mathrm{SctO}}_{2}$ signals, both modalities were interfaced with a multidevice synchronization platform (Moberg Research, Inc., Amber, Pennsylvania, United States), ensuring precise alignment. The data were subsequently saved for offline analysis using MATLAB (MathWorks, Inc., Natick, Massachusetts, United States). For this study, bipolar EEG data from the cross-hemisphere central region electrode pair “C3” and “C4” were specifically selected for analysis.^15^^,^^22^ The EEG underwent preprocessing, beginning with an asymmetric band-pass filter (Parks–McClellan linear-phase finite impulse response filter) to attenuate frequencies below 2 Hz and above 15 Hz.^22^^,^^26^ These filtered signals were then converted into aEEG using the Washington University-Neonatal EEG Analysis Toolbox.^27^

To prepare the aEEG and ${\mathrm{SctO}}_{2}$ data for analysis, a series of preprocessing steps was applied to clean and standardize the signals. Initially, the aEEG data were downsampled to match the sampling rate of the ${\mathrm{SctO}}_{2}$ signals to ensure temporal alignment between the two modalities. Artificial spikes in the aEEG data (typically caused by motion artifacts or electrical interference) were first identified as values exceeding 25  μV. These values were removed and replaced using neighboring values. Both signals then underwent first-order polynomial detrending to eliminate slow, linear trends over time. To suppress noise and short-term fluctuations, a thresholding step was applied. For aEEG, values deviating more than ±5  μV from the fitted trend were adjusted using neighboring data points. For ${\mathrm{SctO}}_{2}$, a wider threshold of ±20% was applied to account for physiological variability in oxygen saturation. Finally, second-order polynomial detrending was used to remove any remaining low-frequency drifts in both signals. These preprocessing steps ensured that the data were clean, continuous, and suitable for subsequent analysis.

### 2.4 Post-Acquisition WTC Analysis

After preprocessing the aEEG and ${\mathrm{SctO}}_{2}$ signals for each neonate, we used a MATLAB-based software package to conduct the WTC analysis.^28^^,^^29^ This analysis applied an analytic Morlet wavelet to process the data. WTC is a robust method in the time–frequency domain that quantifies the relationship between two nonstationary time series. The output results of WTC analysis have two key metrics: the squared cross-wavelet coherence ($R^{2}$) and the relative phase (Δϕ) across different time and frequency scales, without a prior assumption of linearity and stationarity. The original methodology and computational framework were developed for geophysical applications,^28^^,^^29^ but it has been further advanced for the analysis of complex physiological signals with dynamic temporal and spectral features.^14^^,^^15^^,^^22^^,^^30^^,^^31^

#### 2.4.1 Generation of the NVC map

**Fig. 1:**
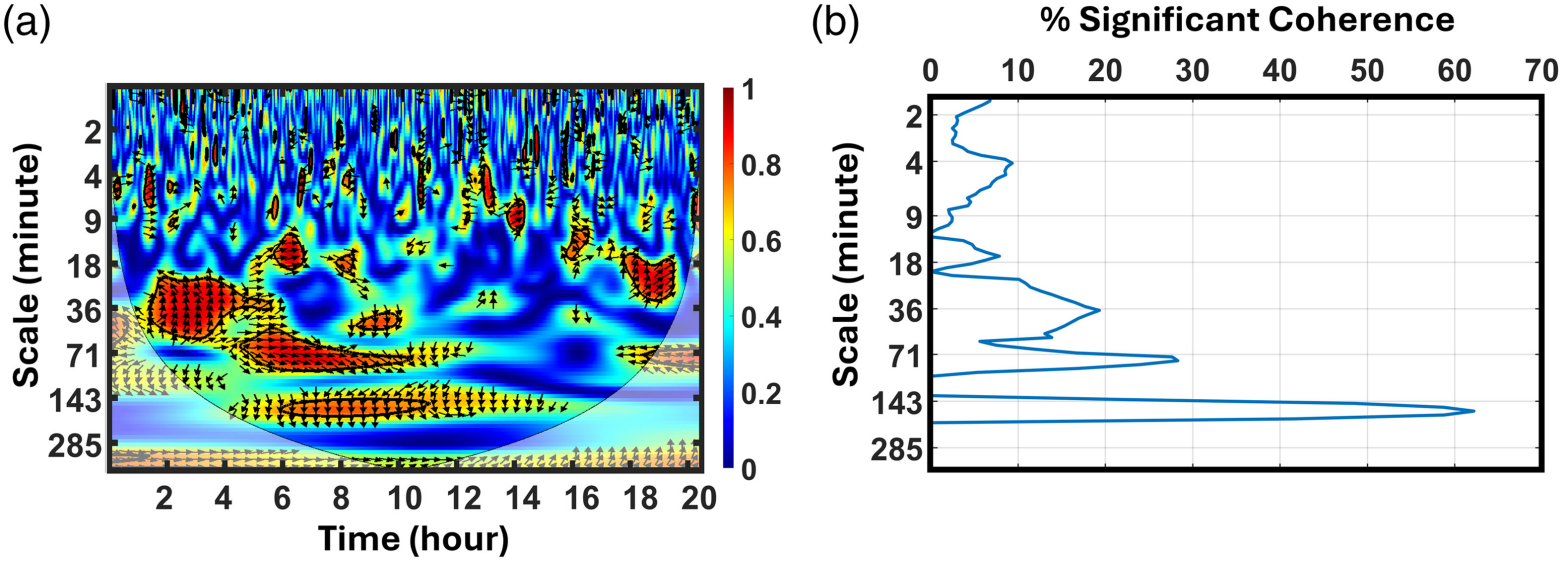
(a) Example of a WTC map obtained from a newborn with a 20-h time duration and a corresponding scale range of 1 to 400 min. The black contours outline the time–frequency (WTC) regions having significant coherence or NVC. (b) The temporal percentage of significant coherence, P(S), across the entire 20-h period across the scale range, matching that in panel (a).

Following our previously published framework,^14^^,^^15^ an NVC map can be generated through WTC analysis [see Fig. 1(a)], displaying coherence values of $R^{2}$ between aEEG and ${\mathrm{SctO}}_{2}$ signals over a 20-h period and across a range of scales (S=1 to 400 min). $R^{2}$ can be conceptualized as a temporally localized correlation coefficient (ranging from 0 to 1) between these two signals. The statistical significance of $R^{2}$ is calculated using a Monte Carlo simulation with a 95% confidence interval. The $R^{2}$ regions that are statistically significant against simulated background noise (p<0.05) are contoured with black curves.^14^^,^^15^^,^^32^ A comprehensive depiction of the degree of synchronization or coherence between neural activity and cerebral oxygenation over time in different wavelet scales (S) or frequency ($f_{\mathrm{wt}}$) can be visualized by this WTC map.^15^^,^^33^ Note that $S=1/f_{\mathrm{wt}}$ when the Morlet base function is used in the WTC calculation.^28^^,^^29^

Furthermore, the percentage of significant coherence, P(S), was quantified as the percentage of time during which the ${\mathrm{SctO}}_{2}$ and aEEG coherence were statistically significant from the noise background (p<0.05). Note that P(S) is a function of the wavelet scale and is calculated across the entire measurement time period (such as 20 h) at each S value within the valid region of the time–frequency space/map, namely, outside of the cone of influence.^28^^,^^29^ Figure 1(a) shows a WTC time–frequency map of a newborn measured in 20 h, whereas Fig. 1(b) presents an example of P(S) corresponding to the WTC map of Fig. 1(a). Figure 1(b) indicates that during 62% of the recording time (e.g., 20 h), this newborn had significant coherence around a scale of 165 min. It also implies that the brain of this newborn had inactive NVC during 38% of the measurement period. This percentage of coherence time varies across different scales between 1 and 400 min, with a coherence scale peak at 165 min.

#### 2.4.2 Time-resolved analysis of NVC

**Fig. 2:**
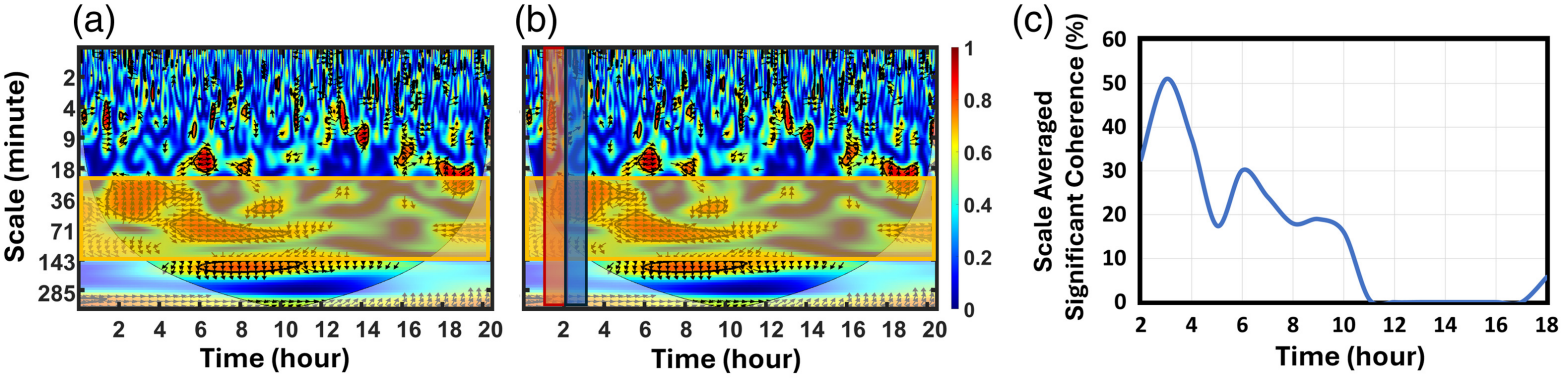
(a) Same WTC map as that in Fig. 1(a) with a yellow horizontal rectangle highlighting a selected scale range of 20 to 150 min for the WTC calculations. (b) Two 1-h windows are outlined by red and blue vertical rectangles with solid lines. Such hourly moving windows are used for dWTC quantifications. (c) Temporal percentages of significant coherence for NVC in each hour were calculated within scales of 20 to 150 min across 20 h of data collection. Note that hour 2 represents the NVC calculated over 1 to 2 h of recording, whereas hour 18 represents the calculated NVC between hours 17 and 18.

As WTC is a time–frequency analysis, it is necessary to select a range of scales or frequencies ($S=1/f_{\mathrm{wt}}$) if one wishes to obtain a temporal evolution of NVC. Accordingly, we selected a scale range of 20 to 150 min [Fig. 2(a)] and then took a 1-h moving window [e.g., red and blue vertical rectangles in Fig. 2(b)] to evaluate scale-selected dynamic changes in significant coherence over 20 h of measurements for the example given [Fig. 1(a)]. Specifically, within each 1-h window, the percentage of pixels within the significant coherence contours over the total number of pixels within the selected time–frequency rectangle was calculated. As the window was shifted hourly, the temporal variation in the percentage of significant coherence was computed within the selected scale range for the respective hours. The final time-resolved significant coherence (trSC in %) value was achieved after completion of the 20-h moving-window calculations. Figure 2(c) shows a scale-selected, hourly derived percentage of trSC through 20 h of measurements starting from hours 2 to 18. Hours 0 to 2 and 18 to 20 were excluded for the dWTC to avoid the edge effect of WTC calculations. In this way, we represented the time-resolved assessment of coherence and captured the temporal evolution of NVC. 

### 2.5 Simulated Real-Time WTC Analysis

To calculate dynamic NVC, we simulated real-time data collection and WTC analysis using a zero-padding strategy. Namely, this approach utilized preprocessed aEEG and ${\mathrm{SctO}}_{2}$ signals over a 20-h duration, with signal segments incrementally revealed to mimic the progression of real-time analysis.

**Fig. 3:**
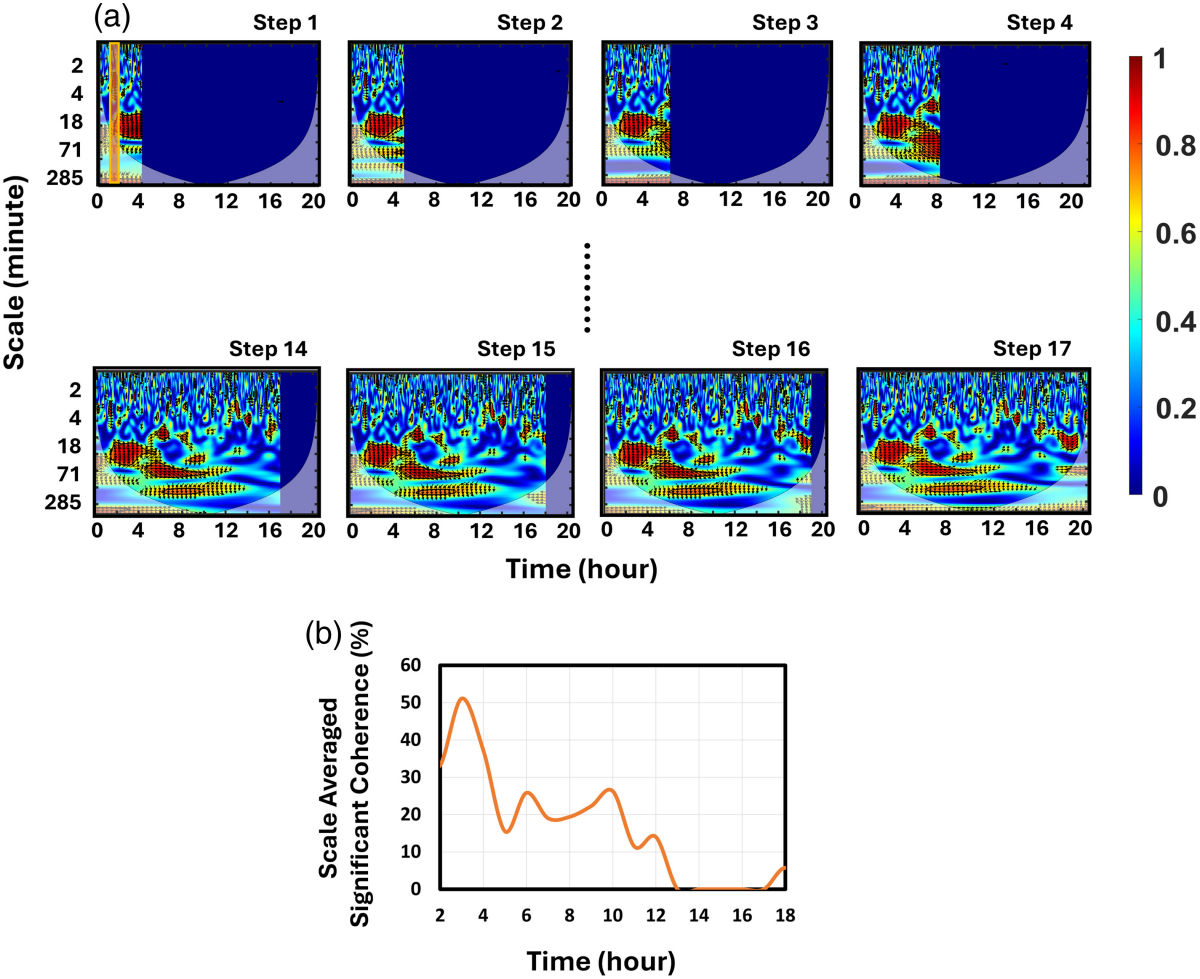
Dynamic WTC (or NVC) analysis using decremental zero-padding to simulate real-time measurements. (a) Data process 1: schematic of the iterative zero-padding approach, where aEEG and ${\mathrm{SctO}}_{2}$ signals are progressively expanded in 1-h increments starting from 4 h. At each step, a corresponding WTC over the entire window is obtained. However, only the percentage of significant coherence corresponding to the time point 2 h prior to the end of the WTC window is calculated (i.e., the time window for which significant coherence was calculated is indicated in step 1 by the vertical yellow rectangle). (b) Data process 2: time-resolved significant coherence (trSC) within the 20 to 150 min scale range, plotted across the 20-h duration based on the sequentially extracted time points from each expanded window.

*Process 1 shown in* Fig. 3(a): *decremental zero padding with hourly steps*. As step 1, the first 4 h of the aEEG and ${\mathrm{SctO}}_{2}$ signals were selected to generate an initial WTC map, whereas both of the signals were zero-padded from hours 4 to 20 [Fig. 3(a)]. The choice of a 4-h time window was intended to minimize the edge effects of WTC, also known as the cone of influence (Refs. 28 and 29). Following step 1, a 1-h window was progressively shifted forward in time, hour by hour, from hours 4 to 20, using decremental zero padding. This approach generated hour-resolved WTC maps, as illustrated in steps 2 through 17 of Fig. 3(a). In this manner, we simulated time-dependent or dynamic WTC maps corresponding to the evolving measurements of aEEG and ${\mathrm{SctO}}_{2}$ signals.

*Process 2 shown in* Fig. 3(b)*: trSC*. For the WTC map obtained in step 1, we followed the same approach described in Sec. 2.4.2. Specifically, we calculated the hourly percentage of significant coherence at hour 2 across the selected scale range (i.e., 20 to 150 min) using the 1-h time window from hours 1 to 2. This yielded the first initial temporal point in the trSC plot within the 20- to 150-min scale range [Fig. 3(b)]. A 4-h window was required to quantify this initial trSC value because the WTC map must extend at least 2 h prior to the time point of interest to ensure reliable time-resolved WTC estimation. The same analysis procedure was repeated for each step of the WTC maps seen in Fig. 3(a), leading to the evolving percentage of significant coherence calculated for the newly updated time points. Figure 3(b) illustrates the resulting curve of the percentage of significant coherence over the entire duration of 20 h, providing a dynamic representation of NVC in the context of real-time simulation. It is important to note that Fig. 3(b) displays data only from hours 2 up to 18 due to the requirement of a minimum 2-h data in advance to ensure the accuracy of real-time trSC calculations.

### 2.6 Statistical Analysis

Demographic and clinical characteristics of the newborns were summarized using descriptive statistics and stratified by treatment group: non-TH (mild HIE) and TH (which included mild–moderate, moderate, and severe HIE). Continuous variables were presented as means with standard deviations or medians with interquartile ranges (IQRs), depending on distribution. Categorical variables were summarized as counts and percentages. Comparisons between the TH and non-TH groups were conducted using Student’s t-test or the Wilcoxon rank sum test for continuous variables and the chi-square ($\chi^{2}$) test or Fisher’s exact test for categorical variables, as appropriate. A linear mixed-effects model was performed to compare the dynamic NVC results of the percentage of significant coherence obtained using the post-acquisition and real-time WTC analysis across all neonates. Specifically, the analysis aimed to determine whether the trend in coherence values over time differs between the two methods, if one method consistently produces different coherence values compared with the other, and if coherence values change significantly over time regardless of the method used. *Post hoc* comparisons were conducted using estimated marginal means derived from the fitted models to assess pairwise differences between the TH and non-TH groups at each hourly time point, separately for the real-time and post-acquisition methods. All statistical analyses were performed using R version 4.2.2.

## 3 Results

### 3.1 Demographics of the Study Cohort

Fifty-five full-term newborns (gestational age: 39 [38, 40] weeks) with mild-to-severe HIE were included. All infants underwent continuous, simultaneous EEG and NIRS monitoring, initiated at 12±6  h of life after parental consent was obtained. TH was initiated at 4±1  h of life in 25 newborns with moderate-to-severe HIE (TH group). Newborns with mild HIE did not receive TH (non-TH group) per standard care; however, five of them progressed between 6 and 24 h of life and subsequently received late TH,^25^ initiated at 15±8  h considered the TH group.

Maternal and neonatal characteristics for the overall cohort, the non-TH group (n=25), and the TH group (n=30) are summarized in Table 1. Overall, 58% of infants were male,^34^ and the mean birth weight was 3.3±0.7  kg. Cesarean section was the mode of delivery in 64% of cases. The TH group had significantly lower Apgar scores at 1 and 5 min and lower umbilical cord pH. The TSS within the first 6 h was significantly higher in the TH group (10 [7, 11]) compared with the non-TH group (4 [3, 5]), indicating severe abnormalities. Such an approach, evaluating the total spectrum of encephalopathy and using TSS as a threshold if replicated in larger studies, could solve the mild HIE definition conundrum. Such a targeted approach to infants who do not meet the current cooling criteria would minimize exposure of low-risk infants to unnecessary interventions and maximize the benefits to those at high risk. As animal studies suggest that less severe insults are the most likely to respond to neuroprotective therapies, it is possible that infants with high TSS scores within the first 6 h of age would be the most likely population to benefit.

**Table 1:** Characteristics of the study cohort.

Characteristics	Overall cohort	Encephalopathy grade		p value
		Non-TH group (mild HIE)	TH group (moderate/severe HIE)	
Total N	55	25	30	
Male: N (%)	32 (58)	16 (64)	16 (53)	0.600
Gestational age (weeks), median [IQR]	39 [38, 40]	39 [38, 40]	39 [38, 40]	0.877
Birth weight (kg), mean (SD)	3.3 (0.7)	3.2 (0.5)	3.3 (0.9)	0.474
Apgar 1-min, median [IQR]*	2 [1, 4]	3 [2, 5]	1 [1, 3]	0.001
Apgar 5-min, median [IQR]*	6 [4, 8]	7 [6, 8]	5 [2, 7]	0.003
Umbilical cord gas pH, mean (SD)*	7.1 (0.2)	7.1 (0.2)	7.0 (0.1)	0.018
Base deficit, median [IQR]	17.1 [14.6, 20.4]	17.1 [14.4, 20.4]	16.8 [14.8, 20.2]	0.949
Total Sarnat score, median [IQR]*	6 [3, 10]	4 [3, 5]	10 [7, 11]	<0.001
Abnormal MRI (global): N (%)	20 (36)	8 (32)	12 (40)	0.739
Maternal race/ethnicity: N (%)				
Caucasian non-Hispanic	2 (4)	1 (4)	1 (3)	0.841
Black non-Hispanic	9 (16)	3 (12)	6 (20)	
Hispanic	41 (75)	20 (80)	21 (70)	
Other non-Hispanic	3 (5)	1 (4)	2 (7)	
Delivery mode: N (%)				
C/S	35 (64)	16 (64)	19 (63)	>0.999
Vaginal	20 (36)	9 (36)	11 (37)	
Maternal risk factors: N (%)				
Hypertension	11 (20)	4 (17)	7 (23)	0.736
Diabetes	3 (5)	3 (12)	0 (0)	0.088
Pre-eclampsia	16 (29)	5 (21)	11 (37)	0.243
Labor complications: N (%)				
Meconium	17 (31)	5 (21)	12 (40)	0.153
Umbilical cord prolapse	2 (4)	1 (4)	1 (3)	>0.999
Placental abruption	4 (7)	1 (4)	3 (10)	0.620
Uterine rupture	3 (6)	1 (4)	2 (7)	>0.999
Maternal chorioamnionitis	17 (31)	6 (24)	11 (37)	0.472
Disposition				
DOL at discharge, median [IQR]*	10 [6, 16]	6 [5, 10]	13 [10, 20]	<0.001
Death prior to discharge, N (%)	2 (4)	0 (0.0)	2 (7)	0.495

Abbreviations: SD = Standard deviation, IQR = Interquartile range, DOL = days of life.

When the normality assumption was violated, the median and IQR were reported, and the Wilcoxon rank sum test was used to assess statistical significance, as appropriate.

* Indicates statistical significance (p<0.05).

The cohort was predominantly Hispanic (75%). Maternal risk factors and labor complications did not differ significantly among groups. Abnormal brain MRI findings prior to discharge were observed in 32% of newborns in the mild group and 40% of those who received therapeutic hypothermia. Infants with mild HIE were discharged earlier than those who received TH (6 [5, 10] versus 13 [10, 20] days). Two newborns with severe HIE died prior to discharge due to redirection of care.

The aEEG data from cross-cerebral central region bipolar EEG (C3–C4) were used to calculate NVC.^15^^,^^22^ NIRS data were recorded at a sampling rate of 0.21 Hz in 50 neonates and at 0.03 Hz in 5 neonates with mild HIE. To assess the impact of this difference in sampling rates, we resampled the 0.21 Hz data to 0.03 Hz for a few subjects and found no statistically significant difference in the calculated percentage of NVC, supporting the comparability of the two sampling rates. Across all study participants, 6% of the time series data per subject was interpolated during artifact removal. All interpolated segments were visually inspected to ensure data quality.

### 3.2 Comparison of Post-Acquisition and Simulated Real-time Analysis

**Fig. 4:**
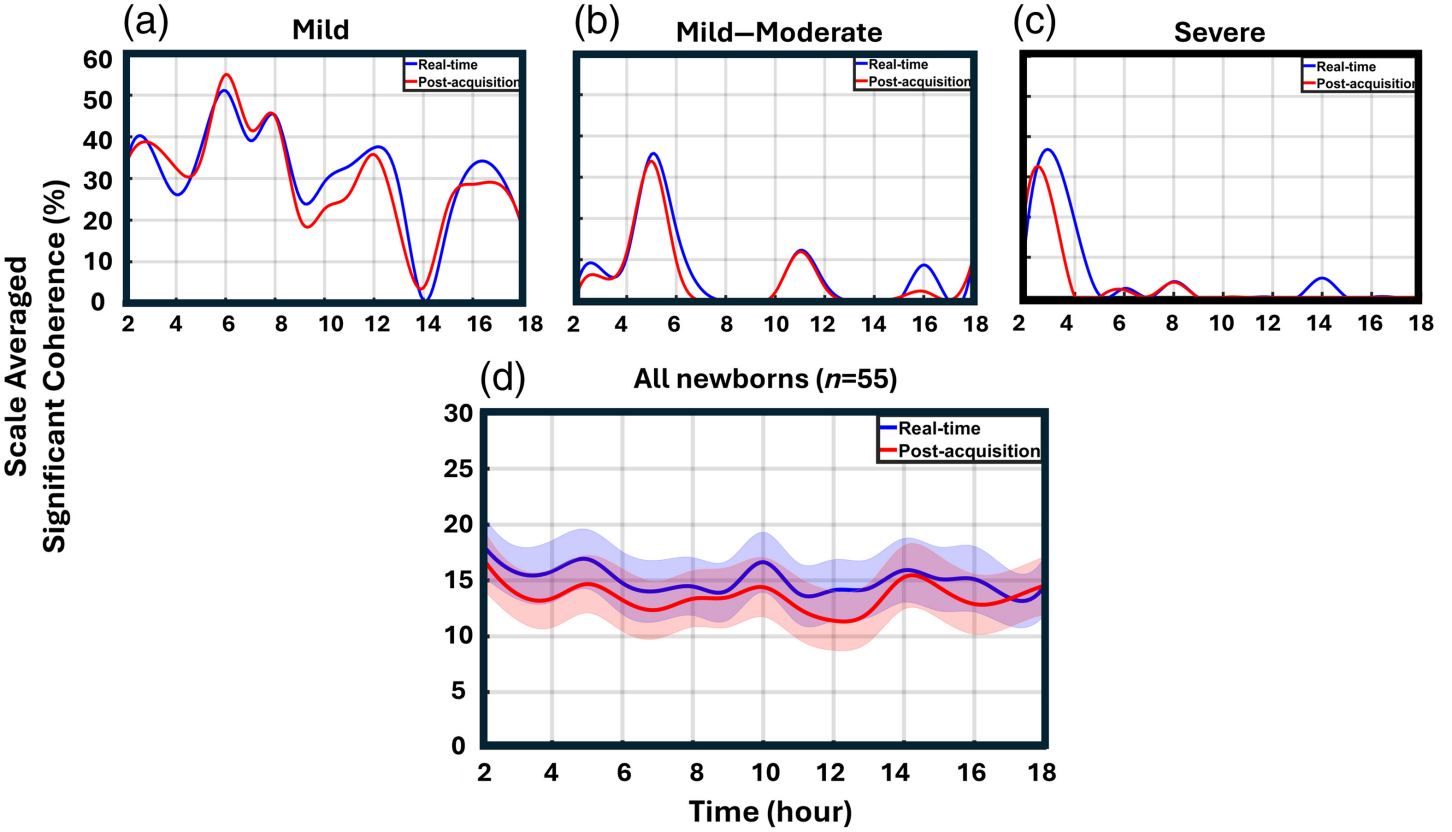
Illustration of real-time versus post-acquisition methods in newborns with HIE. (a), (b), and (c) Cases with mild, mild–moderate, and severe HIE, with total Sarnat scores of 3, 2, and 18, respectively. (d) The comparison between real-time and post-acquisition methods across all newborns (n=55) with HIE.

**Fig. 5:**
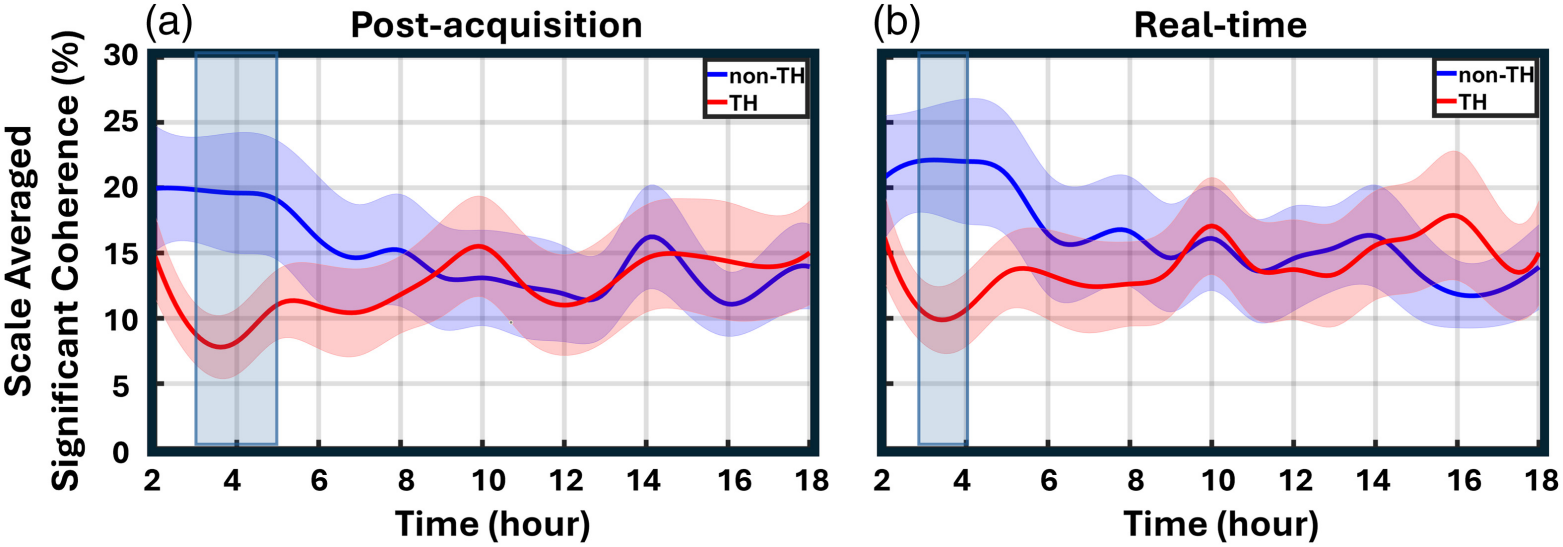
Comparison of NVC between newborns who received TH and those who did not. Post-acquisition (a) and real-time (b) methods, both comparing the non-TH (n=25) versus TH (n=30) groups. The shaded region indicates time points where the percentage of NVC is significantly higher in the non-TH group compared with the TH group.

Both the post-acquisition and real-time (dynamic WTC) methods were applied to analyze NVC data for all 55 neonates (25 mild, 27 moderate, and 3 severe). The results of the percentage of significant coherence over time for three neonates are individually presented in Figs. 4(a)–4(c) for a scale range of 20 to 150 min. Additional example plots comparing post-acquisition and real-time methods for other neonates are provided in the figures of the Supplementary Material. The y-axis can be labeled as time-resolved significant coherence (trSC; %). On visual inspection, the two trSC curves using the post-acquisition and real-time analysis methods exhibit similar temporal patterns for each neonate. For each newborn, two NVC curves were obtained over time, beginning at hour 2 (corresponding to the 1- to 2-h window) and ending at hour 18 (17- to 18-h window). These individual curves were then averaged for each method. Group averages with shaded error bars are presented in Fig. 4(d). A linear mixed-effects model was performed and revealed no significant difference between the two processing methods (estimate = 2.171, 95% CI: −0.847 to 5.189, p=0.159). There was also no significant effect of time (estimate = −0.063, 95% CI: −0.255 to 0.129, p=0.250), nor any interaction between method and time (estimate = −0.071, 95% CI: −0.342 to 0.200, p=0.607). These findings indicate that the temporal trends in significant coherence were comparable for both post-acquisition and real-time WTC analysis approaches. For both post-acquisition and real-time processing methods, group-averaged NVC curves were generated for the TH and non-TH groups, with shaded error bars to illustrate variability across the group (Fig. 5).

For the real-time method, *post hoc* analysis using estimated marginal means revealed significant group differences at specific time points. At hour 3, the TH group had a mean value 12 units lower than the non-TH group (95% CI: −20.20 to −3.82, p=0.004), indicating a statistically significant difference. A similar significant difference was observed at hour 4 (Estimate=−11.60, 95% CI: −19.80 to −3.40, p=0.006). *Post hoc* analysis for the post-acquisition method revealed that the TH group had significantly lower measurements than the non-TH group at hour 3 (Estimate=−11.45, 95% CI: −19.40 to −3.53, p=0.005), indicating a statistically significant difference. Similar differences were observed at hour 4 (Estimate=−11.75, 95% CI: −19.70 to −3.83, p=0.004) and hour 5 (Estimate=−8.33, 95% CI: −16.30 to −0.41, p=0.040). For the real-time method, *post hoc* analysis using estimated marginal means revealed significant group differences at hours 3 and 4, which is within the most critical time for determination and efficacy of cooling within 6 h of the primary insult.

## 4 Discussion

This research demonstrates the feasibility and reliability of real-time dynamic WTC analysis using a zero-padding technique to assess the temporal and frequency-specific dynamics of NVC in neonates with HIE. The results reveal that both the real-time dWTC approach and the traditional post-acquisition method yield comparable temporal trends in coherence percentage curves across all subjects within the scale range of 20 to 150 min. This consistency highlights the robustness of the proposed real-time WTC analysis method, which successfully captures evolving NVC patterns over a 20-h period.

Our analysis revealed that significant differences in NVC values between the TH and non-TH groups emerged as early as hours 3 and 4 using each of the post-acquisition and the real-time methods. These findings highlight the potential of early NVC monitoring to detect physiological differences within the first few hours of life, which may help identify neonates with more severe encephalopathy. Newborns start to receive TH within 6 h for maximal benefit once they qualify. The groups with moderate-to-severe HIE receiving cooling after 6 h of birth had comparable NVC to mild HIE untreated. This potentially will be important to address in new trials of TH for mild HIE as COOLPRIME (NCT04621279). The capacity to measure NVC in real time within the therapeutic window for TH as well as testing the subsequent effect of treatment will impact clinical decision-making and risk stratification in a timely manner.

A clinically significant reduction in death or survival with disability was reported in newborns with moderate-to-severe HIE in multicenter international randomized-controlled trials of TH.^5^^,^^35^ Early clinical trials excluded infants with mild HIE, identified through the modified Sarnat examination, from treatment arms due to potential risks associated with TH and the assumption that outcomes for this group of infants were likely to be favorable.^8^ However, more than half of all newborns with HIE are categorized as mild,^10^ and it has been observed that survivors of mild HIE manifest both acute and chronic neurological impairment. The pre-hypothermia era data indicated that older school-aged children with a history of mild HIE at birth had an increased risk of behavioral dysregulation and attention deficits.^36^^,^^37^ Prempunpong et al.^38^ reported that a large proportion (28/54, 52%) of infants enrolled in the Prospective Research on Infants with Mild Encephalopathy (PRIME) study had abnormalities on aEEG, MRI, or neurological exam at time of discharge from neonatal intensive care. A meta-analysis conducted by Conway et al.^11^ found an abnormal neurodevelopmental outcome among 25% (86/341) of infants with mild HIE based on data from 4 randomized controlled trials and 16 prospective observational cohort studies.

This study demonstrates that when an appropriate WTC scale range (such as 20 to 150 min) is chosen, the real-time WTC analysis method can be used in capturing key neurovascular dynamics. This new analysis framework is particularly important for time-sensitive interventions in neonates with HIE as it allows clinicians to dynamically monitor and respond to changes in NVC. Thus, this new WTC analysis strategy shows promises for improving early detection and intervention, provided that the scale range is carefully selected to capture the relevant neurovascular dynamics.

Das et al.^15^ demonstrated that an NVC cutoff of 10% on the first day of life predicted brain injury observed in MRI on day 5 of life in newborns with mild-to-severe HIE, outperforming the clinical Sarnat examination. Notably, 40% of newborns with mild HIE in studies exhibited abnormal MRI findings. Similarly, Kota et al.^16^ reported that an NVC cutoff of 12% predicted neurodevelopmental outcomes at 2 years of age. Real-time evaluation of NVC during the first day of life could have facilitated timely neuroprotective interventions, potentially improving outcomes.

The primary aim of the current research is to establish the feasibility of implementing NVC monitoring in real time. In our previous work, we demonstrated that NVC can document NVC before and after erythropoietin (Epo) administration in neonates with moderate and severe HIE, and NVC was unchanged following Epo treatment, mirroring the lack of clinical efficacy at 2 years.^39^ Building on this evidence, the real-time NVC approach proposed in this paper may also be applicable to detecting seizure activity that we are actively exploring in ongoing studies, monitoring evolving encephalopathy symptoms, and dynamic changes following neuroprotective intervention. It may also aid in early prediction of HIE severity, brain injury, and long-term neurodevelopmental outcomes during the early critical window within 24 h of life^5^^,^^25^^,^^40^ when therapeutic decisions are most impactful. Future work will aim to define clinically meaningful thresholds for the percentage of NVC during this early period and to characterize temporal patterns that reflect encephalopathy progression, thereby supporting more personalized neuroprotective strategies. Ongoing prospective COOLPRIME (NCT04621279) trial with 18 enrolling institutions will validate the clinical utility of real-time NVC monitoring in neonates with HIE.

There are several methods to calculate aEEG from EEG signals, and no significant differences in NVC results were observed when using three different aEEG algorithms.^22^ Similarly, comparable findings were obtained using both aEEG and processed EEG, making these methods ideal for real-time bedside monitoring.^24^ WTC is frequently used to estimate autoregulation by analyzing the relationship between mean arterial pressure and ${\mathrm{SctO}}_{2}$. The method proposed in the present study has the potential to be adapted for measuring autoregulation in real time in newborns with HIE^30^ and critically ill patients with severe respiratory and/or cardiovascular failure undergoing extracorporeal membrane oxygenation.^31^

The proposed method for real-time NVC estimation shows significant potential but has some limitations. It relies on aEEG and NIRS parameters, and variations in data acquisition protocols, equipment, or algorithms could affect its consistency. However, our earlier findings demonstrated that different aEEG and processed EEG methods produce comparable outcomes, underscoring the robustness of the approach.^22^^,^^24^ The single-center nature of the study may limit the generalizability of the findings, but we have the potential to expand this research (NCT04621279) to multiple sites, including infants with 2-year neurodevelopmental outcomes, to enhance validation and applicability. In addition, WTC measurements can be affected by the cone of influence, but this issue can be mitigated using mirrored signals before and after the data segment. Addressing these limitations in future studies will be crucial for enabling the broader clinical adoption of this method for real-time bedside monitoring of NVC, allowing for the early identification of newborns at risk of progressing to more severe HIE and facilitating timely neuroprotective interventions to improve outcomes.

## 5 Conclusion

The introduction of a real-time analysis framework for assessing dynamic NVC represents a significant leap forward in neonatal care, addressing the critical need for tools that facilitate immediate and continuous evaluation of cerebral hemodynamics and neuronal activity. Such capabilities are particularly vital in neonatal intensive care units, where early detection of abnormalities and timely interventions are crucial to prevent long-term neurological impairments. By delivering near real-time insights, the dynamic NVC approach aligns with the pressing demand for early diagnosis and effective treatment strategies in neonatal care. It holds the potential to transform clinical decision-making, particularly in urgent and time-sensitive scenarios such as therapeutic hypothermia, where interventions must occur within narrow therapeutic windows to mitigate damage and improve outcomes.

## Supplementary Material

10.1117/1.NPh.12.3.035011.s01

## Data Availability

Data and computer code access requests should be directed to the corresponding author and must include a reasonable justification. Data will be provided in accordance with relevant data protection and confidentiality policies.
