# Supplementary material for: Real-time monitoring of wavelet-based neurovascular coupling in neonates with hypoxic ischemic encephalopathy using an hourly time window
Source: Neurophotonics. 2025 Sep 9;12(3):035011. doi: 10.1117/1.NPh.12.3.035011 (PMC12419756; doi:10.1117/1.NPh.12.3.035011)
Supplement: Supplementary file 1 [file NPh_012_035011_SD001.pdf]

Real-Time Monitoring of Wavelet-Based Neurovascular Coupling in Neonates with Hypoxic Ischemic Encephalopathy (HIE) Using Hourly Time Window

Soheila Norasteh[1], Hanli Liu[1], Srinivas Kota[2], Yu-Lun Liu[3], Rong Zhang[4], and Lina F. Chalak[2,*]

[1]Department of Bioengineering, University of Texas at Arlington, Arlington, TX, USA
[2]Division of Neonatal-Perinatal Medicine, Department of Pediatrics, UT Southwestern Medical Center and Children's Medical Center, Dallas, TX, USA
[3]Peter O'Donnell Jr. School of Public Health, UT Southwestern Medical Center, Dallas, TX, USA
[4]Department of Neurology, UT Southwestern Medical Center, Dallas, TX, USA

Supplementary Material

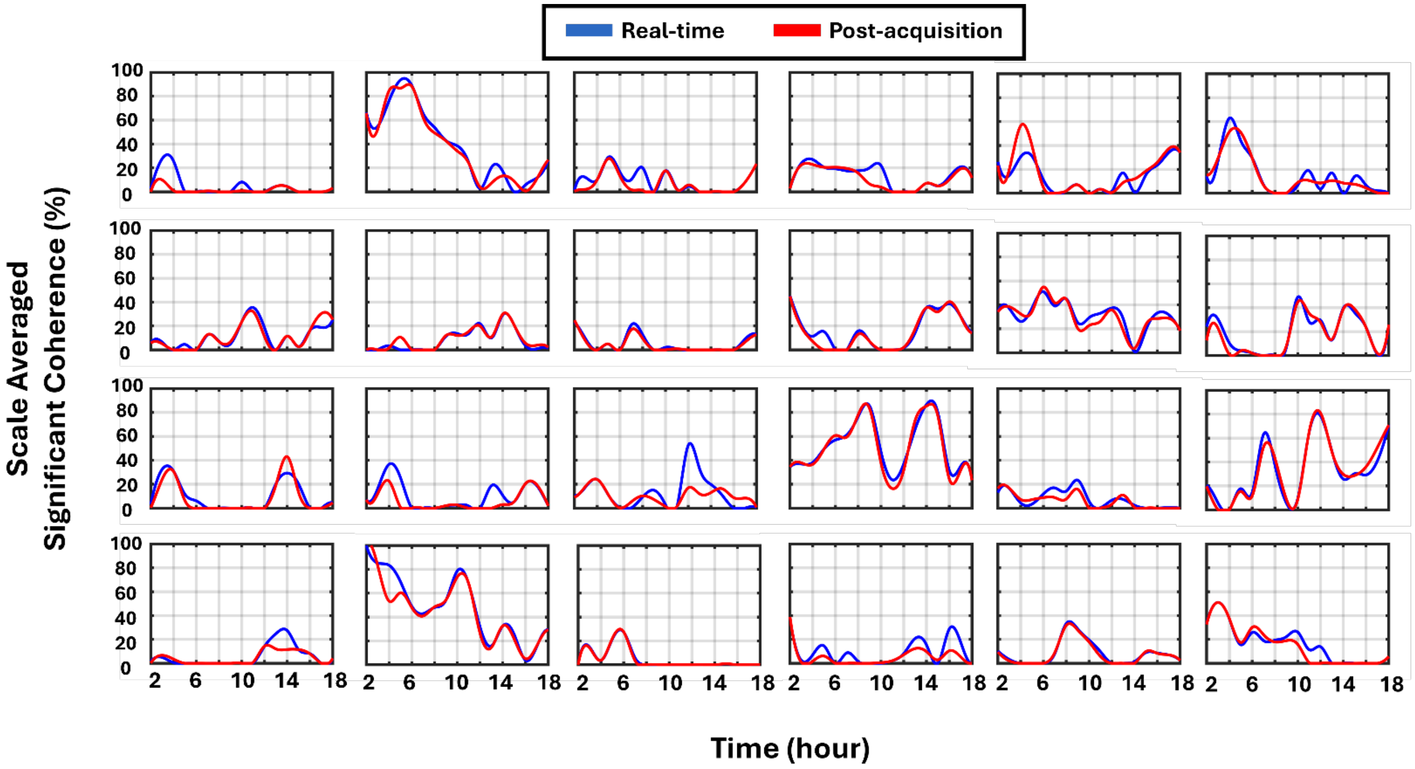

Figure S1. Comparison of the percentage of significant coherence calculated using the post-acquisition method (red curve) and the real-time simulation method (blue curve) for a scale range of 20-150 minutes for 24 newborns with mild HIE.

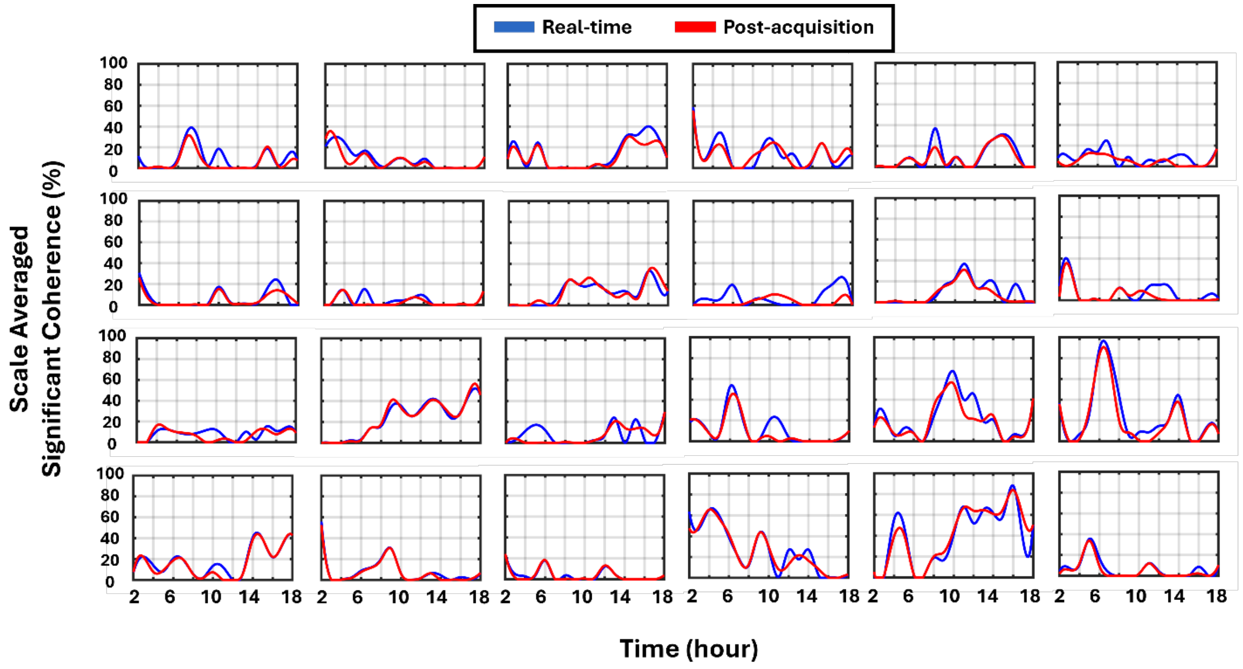

Figure S2. Comparison of the percentage of significant coherence calculated using the post-acquisition method (red curve) and the real-time simulation method (blue curve) for a scale range of 20-150 minutes for 24 newborns with moderate HIE.
